# From cytogenetics to proteogenomics: new horizons in the study of aneuploidies

**DOI:** 10.18699/vjgb-25-37

**Published:** 2025-06

**Authors:** K.S. Zadesenets, N.B. Rubtsov

**Affiliations:** Institute of Cytology and Genetics of the Siberian Branch of the Russian Academy of Sciences, Novosibirsk, Russia Novosibirsk State University, Novosibirsk, Russia; Institute of Cytology and Genetics of the Siberian Branch of the Russian Academy of Sciences, Novosibirsk, Russia Novosibirsk State University, Novosibirsk, Russia

**Keywords:** aneuploidy, chromosomal instability, genomic diversity, mosaicism, dosage compensation, differential gene expression, monoallelic expression, protein degradation, ubiquitin-proteasome system, architecture of interphase nucleus, анеуплоидия, хромосомная нестабильность, геномное разнообразие, мозаицизм, дозовая компенсация, дифференциальная экспрессия генов, моноаллельная экспрессия, деградация белков, убиквитинпротеасомная система, архитектоника интерфазного ядра

## Abstract

Aneuploidy is defined as the loss or gain of a whole chromosome or its region. Even at early stages of development, it usually leads to fatal consequences, including developmental defects/abnormalities and death. For a long time, it was believed that the disruption of gene balance results in pronounced effects at both the cellular and organismal levels, adversely affecting organism formation. It has been shown that the gene imbalance resulting from aneuploidy leads to proteotoxic and metabolic stress within the cell, reduced cell proliferation, genomic instability, oxidative stress, etc. However, some organisms have exhibited tolerance to aneuploidies, which may even confer adaptive advantages, such as antibiotic resistance in pathogenic fungal strains. A significant factor likely lies in the complexity of the tissue and organ organization of specific species. Polyploid organisms are generally more tolerant of aneuploidy, particularly those that have recently undergone whole-genome duplication. This review places special emphasis on the examination of sex chromosome aneuploidies in humans. In addition to primary effects, or cis effects (changes in the quantity of the transcripts of genes located on the aneuploid chromosome), aneuploidy can induce secondary or trans effects (changes in the expression levels of genes located on other chromosomes). The results of recent studies have prompted a reevaluation of the impact of aneuploidy on the structural-functional organization of the genome, transcriptome, and proteome of both the cell and the entire organism. Despite the fact that, in the cases of aneuploidy, the expression levels for most genes correlate with their altered copy numbers in the cell, there have been instances of dosage compensation, where the transcript levels of genes located on the aneuploid chromosome remained unchanged. The review presents findings from recent studies focused on compensatory mechanisms of dosage compensation that modify gene product quantities at post-transcriptional and post-translational levels, alleviating the negative effects of aneuploidy on cellular homeostasis. It also discusses the influence of extrachromosomal elements on the spatial organization of the genome and the changes in gene expression patterns resulting from their presence. Additionally, the review specifically examines cases of segmental aneuploidy and changes in copy number variants (CNVs) in the genome. Not only the implications of their composition are considered, but also their localization within the chromosome and in various compartments of the interphase nucleus. Addressing these questions could significantly contribute to enhancing cytogenomic diagnostics and establishing a necessary database for accurate interpretation of identified cases of segmental aneuploidy and CNVs in the genome.

## Abstract (ru)

Анеуплоидией принято считать потерю или приобретение копии целой хромосомы или ее района. Уже на ранних стадиях развития она, как правило, приводит к фатальным последствиям, включая гибель организма и пороки/аномалии развития. Длительное время предполагалось, что именно нарушение баланса генов приводит к выраженным эффектам как на клеточном, так и на организменном уровне, негативно сказываясь на формировании организма. Было показано, что возникший вследствие анеуплоидии дисбаланс генов индуцирует протеотоксический и метаболический стресс в клетке, ее замедленную пролиферацию, нестабильность ее генома, оксидативный стресс и пр. Однако для некоторых организмов была описана толерантность к анеуплоидиям, которая даже могла способствовать возникновению у них адаптивных преимуществ (например, резистентность к антибиотикам у патогенных штаммов грибов). Вероятно, значимым фактором является сложность тканевой и органной организации особей конкретного вида. К анеуплоидии преимущественно более толерантны полиплоидные организмы и виды, относительно недавно прошедшие полногеномную дупликацию. Особое внимание в обзоре уделено рассмотрению анеуплоидий половых хромосом человека. Помимо первичных эффектов или цис-эффектов (изменение количества транскриптов генов, находящихся на анеуплоидной хромосоме), анеуплоидия может вызывать вторичные или транс-эффекты (изменение уровня экспрессии генов, расположенных на других хромосомах). Результаты исследований последних лет заставили по-новому взглянуть на влияние анеуплоидии на структурно-функциональную организацию генома, транскриптом и протеом как клетки, так и целого организма. Несмотря на то что при анеуплоидии уровень экспрессии для большинства генов коррелирует с измененным числом копий генов в клетке, были описаны случаи дозовой компенсации, при которой уровень транскриптов генов, расположенных на анеуплоидной хромосоме, оставался неизменным. В обзоре приводятся результаты последних исследований, посвященных изучению компенсаторных механизмов дозовой компенсации изменения количества продуктов генов на посттранскрипционных и пострансляционных уровнях, снижающих негативный эффект анеуплоидии на гомеостаз клетки, а также влиянию экстрахромосом на пространственную организацию генома, изменению паттернов экспрессии генов вследствие ее наличия. Кроме того, отдельно обсуждаются варианты сегментных анеуплоидий и изменения числа копий участков генома. Рассмотрено не только значение их состава, но также его локализация в хромосоме и в разных компартментах интерфазного ядра. Решение поднятых вопросов может внести большой вклад в совершенствование цитогеномной диагностики и в создание необходимой базы данных для корректной интерпретации выявленных случаев и сегментной анеуплоидии, и варьирующих по числу копий участков генома.

## Introduction

The loss or gain of a copy of a whole chromosome or its part is referred to as aneuploidy (Tang, Amon, 2013). However, whole-genome sequencing and microarray-based comparative genomic hybridization have dramatically increased our understanding of the variation of the human genome; the view of variations in the copy number of genomic regions has become ambiguous (Pinkel et al., 1998). A high level of polymorphism was identified, and variations in the copy number of certain genomic regions (CNV, Copy Number Variant) quite often represented variants of normal genetic diversity. Unfortunately, the principle of describing the human genome based on a separate assembly of the haploid set often does not allow us to give an unambiguous answer to the question of what a particular case of CNV represents. It might be possible to distinguish between normal genomic diversity and its pathological variants more clearly in the future thanks to the generation of a human pangenome (Liao et al., 2023; Miga 2024). However, at present, it is frequently not possible to determine whether a polymorphism is a normal or pathological variant. CNV is the difference in the copy number of DNA segments found by comparing an individual genome to the reference human genome assembly, which is identified through cytogenomic analysis methods. In contrast to CNV, segmental aneuploidy is more frequently associated with a pathogenic effect, as it has a larger size and typically leads to chromosomal changes that can be detected using cytogenetic methods.

In this review, we will consider CNV as one of three types of segmental aneuploidy, differing in the size and structural organization of the corresponding region of the genome: 1) whole-chromosome aneuploidy – aneuploidy of the entire chromosome; 2) segmental aneuploidy – a change in the copy number of large regions of the genome, detected using classical cytogenetic methods; 3) CNV – a change in the copy number of a region of the genome of 1 thousand base pairs (Dürrbaum, Storchová, 2016). The distinction between these aneuploidy types can occasionally be very arbitrary, and they can also be categorized as distinct aneuploidy types at the same time. For instance, aneuploidy in a chromosomal region is caused by the presence of a small supernumerary marker chromosome in humans

Aneuploidies on an entire chromosome are the result of errors in chromosome segregation. The main cause of these mistakes is the absence or insufficient cohesion of sister chromatids, defects in spindle formation in meiosis or mitosis (multipolar spindle, merotelic kinetochore attachment), and errors in cell cycle checkpoints (Thompson et al., 2010). Segmental aneuploidies often result from the formation of unbalanced gametes in carriers of inversions and balanced translocations. They, like CNVs, can arise due to errors in DNA replication and repair, leading to deletions or amplifications of DNA sequences and structural chromosomal rearrangements (Colnaghi et al., 2011). An abnormal number of chromosomes in the zygote leads to constitutive aneuploidy, the state where all cells are aneuploid. The occurrence of aneuploidy at later stages of organism development leads to somatic mosaicism, which may not have a pathological effect. For example, in some human tissues (brain, liver), a significant number of aneuploid cells are normally detected in the absence of a negative effect on the normal function of these organs (Rehen et al., 2005; Duncan et al., 2012). In this work, we will separately consider the aforementioned variants of aneuploidy, starting with whole-chromosome aneuploidy.

## Constitutive whole-chromosome aneuploidy

Both the complexity of the tissue and organ organization and the peculiarities of the structural and functional organization of the genomes of species belonging to different taxa can cause fundamental differences in the frequency and manifestation of constitutive aneuploidy in different eukaryotic species. Wholechromosome aneuploidy causes developmental defects, which are frequently severe and fatal, in the majority of species. In some organisms, it may nevertheless represent a variation of the norm. By excluding species with microchromosomes in their karyotypes, we may conclude that whole-chromosome aneuploidy results in gene imbalance, which alters the expression of genes located on the aneuploid chromosome. It is widely accepted that this genetic imbalance affects the development and fitness of an organism at the cellular and organismal level (Torres et al., 2007; Williams, Amon, 2009; Rutledge, Cimini, 2016). The transcriptome studies of aneuploids revealed that the expression levels of genes located on euploid chromosomes also changed, in addition to the number of transcripts of genes directly associated with the aneuploid chromosome (Letourneau et al., 2014; Dürrbaum, Storchová, 2016). Even when the copy number of gene transcripts in a cell changes – for instance, genes with a changed copy number – the amount of their protein product may remain unchanged, which makes evaluating the effect of aneuploidy exceedingly challenging (Muenzner et al., 2024).

Human aneuploidy

In humans, all constitutive variants of autosomal aneuploidy, with the exception of the most common trisomies of autosomes 13, 18, and 21 (Tr13, Tr18, and Tr21, respectively), lead to embryonic mortality. Trisomies of chromosomes 13, 18, and 21 lead to serious developmental abnormalities and are associated with certain clinical phenotypes: Patau syndrome (Tr13), Edwards syndrome (Tr18), and Down syndrome (Tr21) (Lejeune et al., 1959; Edwards et al., 1960; Patau et al., 1960).

Sex chromosome aneuploidies are characterized by different clinical features and outcomes. The most common syndromes are Turner (45,X), Klinefelter (47,XXY), trisomy of the X chromosome (47,XXX), and disomy of the Y chromosome (47,XYY) (Berglund et al., 2020), demonstrating high phenotypic variability with a wide range of clinical manifestations. Clinical phenotypes of patients with various variants of sex chromosome aneuploidies (45,X, 47,XYY, 48,XYYY, 48,XXYY, 49,XXYYY, mos 46,XY/47,XYY, 48,XXYY, 49,XXXYY, 47,XXX, 48,XXXX, 49,XXXXX, and 47,XXY) are described in the atlas of K. Jones et al. (Jones et al., 2022). It is worth noting that monosomy of the X chromosome (45,X) in 99 % of cases leads to the death of the embryo in the early stages of development; a small percentage of the embryos survive, which is probably linked to the mosaic form of the karyotype (Gravholt et al., 2019).

In humans, males are haploid for almost all X-linked genes, which suggests a more stringent natural selection of X chromosome variants during evolution in comparison with autosomes based on the presence of pathogenic gene variants and genes, a change in the copy number of which leads to developmental abnormalities. When considering variants of X-chromosome aneuploidy, one should mention inactivation of one of its copies (XCI, X-Chromosome Inactivation). However, in early human embryogenesis, in the cells of the trophectoderm and inner cell mass, both X chromosomes remain active (Deng X. et al., 2014). In humans, XCI is incomplete, with about 20–25 % of genes remaining active. On the one hand, the result of incomplete XCI can be considered as segmental aneuploidy; on the other hand, the inactivated X chromosome is a heterochromatic extrachromosome, the presence of which can lead to a change in the pattern of the entire cellular transcriptome through epigenetic changes (Deng X. et al., 2014). XCI occurs randomly; the mechanisms by which copies of the X chromosome are selected to be inactivated are unknown. The consequence of such inactivation is the emergence of mosaicism in the expression of allelic variants of genes (i. e., unequal expression of parental alleles) associated with the X chromosome (Werner et al., 2024). Random XCI results in approximately half of the cells having the paternal X chromosome inactivated and the other half having the maternal X chromosome inactivated. However, in some cases, unequal XCI may occur, with different tissues having different ratios of cells with inactivated maternal or paternal X chromosomes. Disturbances in equiprobable Xchromosome inactivation (e. g., a mutant allele of an X-linked gene is expressed in most cells) can lead to the development of X-linked diseases (Minks et al., 2008).

About 12–15 % of X-linked genes remain active in all cells, while for another 8–10 % of X-linked genes, transcription is observed only in some cell types (Carrel, Willard, 2005; Balaton et al., 2015). Altered transcription levels, in addition to mRNA, were also detected for non-coding RNA genes (including microRNA, lncRNA, and circular RNA). The expression level of genes that are not subject to inactivation varies widely (10–95 %) in different cell types. Mosaic aneuploidies of sex chromosomes deserve special mention. Since samples of patients’ peripheral blood are most often used for cytogenetic analysis, it is extremely problematic to assess the level of mosaicism of sex chromosomes in different tissues. However, even in the blood of such patients, more than 30 % of mosaic variants were detected for 45,X and 47,XXX karyotypes and a lower level of mosaicism for 47,XXY and 47,XYY (6–7 and 11 %, respectively) (Gravholt et al., 2019; Pavlicek et al., 2022; Tallaksen et al., 2023). The question of to what extent the imbalance in gene copy number is corrected at the proteome level in sex chromosome aneuploidies remains open.

## Whole-chromosome aneuploidy in different species of eukaryotes

The negative impact of aneuploidy manifests itself at both the cellular and organismal levels. In most species of eukaryotes, it leads to developmental abnormalities, diseases, and nonviability. However, the frequency of aneuploidy and its effect on the host phenotype can vary greatly among representatives of different taxa. In mammals, autosomal aneuploidies have a pronounced negative effect, including fatal developmental abnormalities. In addition to humans, a high frequency of aneuploidy has been described in frozen embryos and piglet embryos with developmental defects and in calf embryos obtained in vitro. Sex chromosome monosomies have been identified in sterile sheep and cattle (Bouwman et al., 2023). Genome imbalance leads to proteotoxic and metabolic stress in the cell, slow proliferation, genomic instability, oxidative stress, etc. (Stingele et al., 2012).

However, in some species from a number of taxa, tolerance to aneuploidy has been revealed, for example, in plants – salsify, Tragopogon miscellus (Chester et al., 2012); in fungi – Saccharomyces cerevisiae, Candida albicans (Rustchenko, 2007; Kvitek et al., 2008); in protozoa – Leishmania, Giardia, Trypanosoma (Sterkers et al., 2010); and among flatworms – some representatives of the genus Macrostomum (Zadesenets et al., 2020). Moreover, in aneuploids of some species, an unbalanced karyotype may likely contribute to adaptation to various environments. For example, in a number of pathogenic yeasts, aneuploidy leads to the formation of genomic diversity and the emergence of antibiotic resistance (Pavelka et al., 2010).

Karyotypes of protozoans of the genus Leishmania (the causative agent of leishmaniasis in mammals, including humans) contain from 34 to 36 chromosomes, and a number of studies have shown that aneuploid variants predominate among them (Lachaud et al., 2014). Moreover, an ama- zing feature in the form of constitutive mosaic aneuploidy was revealed in L. major: individuals of the same line, even having the same clonal origin, are mosaic and contain mono-, di-, and trisomic cells on different chromosomes (Sterkers et al., 2011, 2012). Mosaic aneuploidy was later identified in other Leishmania species (Lachaud et al., 2014). In addition to pronounced genotypic and karyotypic diversity, Leishmania is characterized by maintaining high genetic heterogeneity in a population consisting of homozygous individuals. The authors believe that genomic variability, due to the high plasticity of the karyotype, provides phenotypic diversity and is an adaptive mechanism of Leishmania to environmental changes during a complex life cycle (Sterkers et al., 2012).

An unusual variant of aneuploidy was discovered in natural populations and laboratory lines of free-living flatworms of the genus Macrostomum (Zadesenets et al., 2016, 2020). The genomes of M. lignano, M. janickei, and M. mirumnovem arose due to a recent whole-genome duplication followed by intensive reorganization of the duplicated genome (chromosomal fusions, inversions, indels, etc.) (Zadesenets et al., 2020, 2023; Zadesenets, Rubtsov, 2021). In these species, the karyotype evolution involved the fusions of all ancestral chromosomes into one large chromosome. In M. lignano, the presence of aneuploids with tri- and tetrasomy on a large chromosome, exhibiting no phenotypic and reproductive features, was recorded (Zadesenets et al., 2016). The karyotypic variation in M. mirumnovem was so high that a specific nomenclature for the species’ chromosomes had to be established. A hypothetical basic karyotype had to be introduced in order to apply the standards for characterizing karyotypes that are recognized in modern cytogenetics (Zadesenets et al., 2020). A large chromosome with extensive paralogous regions that were highly homologous to the chromosomes of the ancestral set was linked to the whole-chromosome aneuploidy observed in the Macrostomum species (Zadesenets et al., 2017a, b).

A distinct, less noticeable manifestation of aneuploidy in organisms with an increased genome ploidy is worth discussing separately, in addition to species with a recent whole-genome duplication. Comparing the effects of aneuploidy across species reveals its species specificity, which may be related to varying levels of tissue and organogenesis complexity.

## Model systems to study aneuploidy

The presence of species tolerant to aneuploidy would seem to facilitate a simple and effective establishment of experimental models for its study. Indeed, numerous studies performed on aneuploid yeast strains have significantly expanded the fundamental knowledge of the causes and consequences of the effect of aneuploidy on the genome, transcriptome, and proteome of the cell (Torres et al., 2007; Pavelka et al., 2010; Torres, 2023). Moreover, mechanisms for correcting imbalances in gene dosage have been proposed.

In humans, modeling of aneuploidies has naturally been limited to experiments with cell cultures obtained from patients with aneuploidies and/or the creation of aneuploid cells using chromosome engineering methods (MMCT, Microcell- Mediated Chromosome Transfer; targeted chromosome elimination with Cre/loxP, CRISPR/Cas9; induction of CIN) (Fournier, Ruddle, 1977; Thomas et al., 2018; Leibowitz et al., 2021; Zhang X.M. et al., 2022; Truong et al., 2023).

Whole-chromosome aneuploidy in cells cultured in vitro

Cell cultures and lines maintained in vitro are tolerant of aneuploidy. To identify the effect of aneuploidy, their proliferative potential is usually assessed. Whole-chromosome monosomy is rare in cell lines. Note that some authors believe that monosomies, in contrast to whole-chromosome and segmental tri- and tetrasomies, less often lead to chromosomal instability (CIN, Chromosomal Instability) (Taylor et al., 2019). In vivo, monosomies are most often associated with hematological malignancies; monosomies on the arms of some chromosomes are also associated with malignant neoplasms (deletion of 1p – neuroblastoma, 3p – lung cancer, 7q or entire HSA7 – myeloid leukemia) (Taylor et al., 2019). This is likely due to loss of heterozygosity for tumor suppressor genes; for example, deletion of 17p in many tumors is associated with the loss of a copy of the TP53 gene in the absence of its normal allele on the homologous chromosome (Chundury et al., 2021).

The problem of loss of heterozygosity should probably be considered in detail separately, taking into account the possibility of obtaining and maintaining haplodiploid cell cultures. For example, sequencing the genome of cells from one of these cultures allowed for the complete assembly of its haploid set from telomere to telomere (T2T-CHM13), including extended regions of heterochromatin (Nurk et al., 2022). The result was the announcement of the successful completion of the human genome sequencing program (Nurk et al., 2022).

Aneuploid cells in vitro typically exhibit a reduced proliferation rate. Taking into account that they undergo excessive protein synthesis, some of which can be leveled by the ubiquitin- proteasome system, such a slowdown in proliferation seems natural but not critical for obtaining and maintaining cell cultures in vitro. At the same time, a decreased rate of cell proliferation during the development of the organism at various stages of ontogenesis can be critical and lead to serious disorders

It should be noted that when cultivating cell lines in vitro, as in cells at an early stage of tumorigenesis in vivo, genome doubling (WGD, Whole Genome Duplication) can occur, leading to its tetraploidization. Subsequently, these cells, due to a reduction in the number of chromosomes due to tolerance to chromosome segregation errors in mitosis, become aneuploid (hypotetraploid). This may subsequently induce additional genomic instability. Consequently, for cells to simply proliferate, it is not necessary to maintain a balance in the number of chromosomes; moreover, when they overcome the proliferative barrier (the Hayflick limit) and become malignant, it is often accompanied by CIN. In this case, aneuploid cells are better adapted to environmental conditions and proliferate faster. However, rapid proliferation does not ensure coordinated behavior of cells in the organism. Rather, it leads to the formation of various developmental abnormalities, for example, pathologies in histo- and organogenesis.

## Effect of gene dosage on the transcriptome in aneuploidy

The effect of aneuploidy on gene expression has been studied in a variety of experimental models, both cell lines and model organisms. Unfortunately, most studies only assessed the number of transcripts of differentially expressed genes (DEGs). The effect of aneuploidy on the expression of genes localized directly on the chromosome with an altered copy number has been proven in yeast (et al., 2007; Torres et al., 2007), Arabidopsis (Huettel et al., 2008; Sheltzer et al., 2012), maize (Birchler et al., 2013), as well as for mouse (Williams et al., 2008) and human (Nawata et al., 2011; Stingele et al., 2012) cell lines. It is worth noting that aneuploid models included variants of the presence of additional copies of chromosomes and not the loss of one of the copies, i. e., tri- and tetrasomy, not monosomy

To date, experiments conducted in cell cultures and aneuploid model organisms have shown that aneuploidy may have a broader effect on gene expression than previously thought. In addition to the primary cis-effects (changes in the level of transcripts of genes located on the aneuploid chromosome), secondary trans-effects (changes in the expression level of genes located on other chromosomes) were identified (Sheltzer et al., 2012; Birchler, 2013; Dürrbaum, Storchová, 2016).

In in vivo models, the trans effect of aneuploidy on gene expression was first described in maize (Guo, Birchler, 1994). Later, using the example of various cellular and organismal model systems, it was shown that in aneuploidy, the list of DEGs is not limited to the genes of aneuploid chromosomes and includes a significant number of genes from euploid chromosomes. This phenomenon was called the aneuploidyinduced transcriptional response (Sheltzer et al., 2012). Trans effects of aneuploidy have been identified in aneuploid cells of yeast, mice, and humans. In yeast, the trans effect of aneuploidy affects about 5–7 % of genes. When comparing euploid human fibroblasts and fibroblasts with trisomy 21, about 88 % of DEGs are not associated with chromosome 21 but are distributed on other chromosomes (Sullivan et al., 2016). In Turner and Klinefelter syndromes, more than 75 % of DEGs were identified in autosomes, while in carriers of karyotypes 46,XXX and 47,XYY, less than 30 % of DEGs were autosomal (Raznahan et al., 2018). The extent to which trans effects of aneuploidy occur varies among species, and the underlying mechanisms are still poorly understood (Li R., Zhu, 2022).

Thus, the physiological and phenotypic effects of aneuploidy may be associated either directly with changes in the copy number of genes located on the aneuploid chromosome or indirectly with changes in the expression of many genes on euploid chromosomes. The result may be additive or synergistic expression and functional effects at the transcriptional and/or posttranscriptional levels (Pavelka et al., 2010). This is consistent with the nonlinear nature of gene dosage effects that determine subsequent biochemical processes in the cell (Veitia et al., 2013; Pires, Conant, 2016). Although many of the biological effects caused by aneuploidy are consistent with the gene dosage balance hypothesis (Birchler, Veitia, 2012; Veitia, Potier, 2015), it is worth considering the impact of the presence of extra chromosomes on the spatial organization of the nucleus and potentially on the genome-wide transcriptional activity of a wide variety of genes

Separately, it is worth noting that often in studies of the effect of aneuploidy on the transcriptome, non-isogenic lines are used when analyzing DEGs (especially in studies conducted on human cells), which, when conducting a comparative analysis, introduces additional difficulties for correctly assessing the contribution of aneuploidy and the existing genetic diversity. The use of isogenic lines, differing only in the presence of an additional chromosome, could significantly increase the efficiency and reliability of the analysis. Such lines can be obtained by cloning mosaic samples. An alternative approach currently implemented is the comparison of transcriptomes and genomes of individual cells obtained from mosaics based on chromosomal aneuploidies (Wang S. et al., 2024).

Note that the effect of aneuploidy on one chromosome on the cell transcriptome as a whole significantly complicates the analysis and assessment of the effect of aneuploidy. Among the genes listed in the OMIM database (Online Mendelian Inheritance in Man, https://omim.org), only a part of them showed a pathogenic effect when their copy number changed, but the secondary effect of aneuploidy may be an extremely important component of its total pathogenic effect. Nevertheless, it is logical to expect that the more dosage-sensitive genes and genes encoding transcription factors, peptides, proteins, and small RNAs that affect the transcriptional activity of many genes there are in a given chromosome, the stronger the change in the transcriptome and disturbance of homeostasis in the cell, and the more pronounced the pathologies observed during histo- and organogenesis.

## Possible outcomes of gene copy number alterations in individual cells

Monoallelic expression in individual cells should be taken into consideration when analyzing transcriptional changes caused by aneuploidy. In contrast to the data of the singlecell transcriptomes, the gene expression patterns obtained earlier represented averaged data and did not accurately reflect the real gene expression in single human cells. Studies have revealed variability in monoallelic expression for most autosomal genes and gradations in gene expression during parent-of-origin imprinting and X-chromosome inactiva-tion (Borel et al., 2015; Santoni et al., 2017; Garieri et al., 2018). The latter is likely a result of the stochastic and pulsed nature of transcription, in which transcription of each copy of a gene, including its allelic variants, is independently regulated and determines the monoallelic expression of most autoso- mal genes in a significant proportion of cells (Reinius, Sandberg, 2016; Larsson et al., 2019). Thus, despite aneuploidy, in some cells, transcription may occur from one copy of the gene, but at the same time, there will also be cells with transcription from a larger number of its copies. In cells with trisomy, there is an increase in the proportion of cells with simultaneous transcription from two or more copies of the gene, leading to an increase in the number of transcripts by one and a half times when analyzing the cell pool. Moreover, the picture may differ for different genes in one cell, creating a large diversity in the transcriptome of individual cells (Ramsköld et al., 2024).

Some studies have been devoted to the study of transcriptional bursting in individual cells, in which the frequency of transcriptional bursting (the time between acts of transcriptional bursting), its intensity (the number of transcripts synthesized in one act), and the stability of the synthesized mRNA were assessed (Deng Q. et al., 2014; Stamoulis et al., 2019; Larsson et al., 2021; Ramsköld et al., 2024). In this paper, we only note that the pulsed transcription of different copies of genes in a cell is independent, and at a sufficiently low frequency of the transcription act in a cell containing three copies of a gene, it can occur from one or several copies of the gene (Larsson et al., 2021). In most diploid cells, the expression of only one allele is predominant (monoallelic expression) (Stamoulis et al., 2019), while in a triploid cell, different transcript variants can be formed due to mono- or biallelic gene expression (Larsson et al., 2021). The stochastic determination of the transcription pattern and its time results in a distribution of cells based on the level of transcripts from various copies of the gene. Among cells with trisomy, the distribution includes cells with transcription from one copy, from two, and, rarely, from three copies, which provides an average value of the number of transcripts corresponding to a transcript level one and a half times higher than in a diploid cell.

Therefore, the concept of pulsed transcription assumes variability in the level of transcripts within the cell, as well as a high level of variability in the level of transcripts in aneuploidy. This is characterized by the appearance of cells with a high transcript content, the ability to select cells based on the number of transcripts of the corresponding genes, and the reproduction of variability in the number of transcripts in each subsequent generation of cells. Negative selection of cells by a high transcript level for dosage-dependent genes can lead to a delay of cell cycle progression or even induction of apoptosis. In other words, during ontogenesis, aneuploidy causes a continuous loss of cells involved in the formation of new tissues and organs. In some instances, the instability of the epiblast’s development and changes in the development of the hypoblast and trophectoderm are evident at the early stages of development in human embryos with trisomy (Wang S. et al., 2024).

## Dosage compensation at the transcriptome and proteome levels

Despite the fact that in aneuploidy the expression level for the majority of genes correlates with the altered number of gene copies in the cell, there have been instances of dosage compensation where the level of gene transcripts of genes on the aneuploid chromosome remains constant (Guo, Birchler, 1994; Birchler et al., 2001; Hose et al., 2015; Gasch et al., 2016). Some studies have shown that in aneuploidy, transcriptional dosage compensation may be provided by autoregulation of gene expression, suppression of mRNA translation, and mRNA decay. For example, in wild yeasts with an additional copy of chromosome 12, autoregulation (overproduction of a certain protein reduces the transcription of its gene) of the RPL15A and RPL22A genes encoding ribosomal proteins leads to their dosage compensation (Hose et al., 2015). The increased expression of genes encoding certain microRNAs (for example, miR-155) and localized on human chromosome 21 in Tr21 may lead to dosage compensation of genes localized on this chromosome or affect the expression level of genes on other chromosomes. For example, an increase in miR-155 can suppress the expression of the transcriptional regulator BACH1 located on chromosome 21 (Li R., Zhu, 2022).

A pronounced effect of post-translational dosage compensation has been described in aneuploids from natural isolates and laboratory strains of S. cerevisiae (Muenzner et al., 2024). Despite the fact that 20 % of the studied natural isolates were stable aneuploids, similar aneuploid laboratory-engineered strains were less stable. The transcriptomic profiles of the corresponding pairs of natural isolates and laboratory strains were similar, but while approximately 70 % of proteins encoded on aneuploid chromosomes were corrected to normal levels in natural aneuploid isolates, such a correction in laboratory strains was described for less than 50 % of such proteins. Moreover, if a decrease in the excess amount in laboratory strains was mainly observed for complex protein complexes, then in natural aneuploid isolates, the decrease in the excess amount of proteins affected all classes of proteins (Storchová, 2024). An increased level of ubiquitinylation was detected for proteins encoded on aneuploid chromosomes, and their abundance was reduced via the ubiquitin-proteasome system (UPS, Ubiquitin Proteasome System) (Muenzner et al., 2024).

Therefore, in yeast, the ubiquitin-mediated proteasomal degradation system plays an important, and possibly key, role in maintaining the balance of the proteome of an aneuploid cell (Storchová, 2024). The stability of natural aneuploid isolates of S. cerevisiae suggests that in their genome, there is an adaptation to the presence of an additional chromosome, or there is a selection of a genome variant in which aneuploidy not only does not have a negative effect but even has a positive adaptive effect. In addition to the UPS, other proteolytic mechanisms for correcting the proteome (autophagic-lysosomal system, calpain, and caspase enzymes) exist in the cell to regulate protein homeostasis (Noormohammadi et al., 2018). For example, during proteotoxic stress in aneuploid human cells, the transcription factor TFEB is activated, which regulates the expression of genes involved in the autophagic-lysosomal pathway for the degradation of excess protein aggregates, and an additional mechanism for correcting the abundance of protein products in the cell is triggered (Santaguida et al., 2015).

Obviously, the idea of the pathogenic effect of aneuploidy, caused by a single disturbance in the balance of gene copies localized on the aneuploid chromosome, is too simplified. For instance, clinical manifestations with Tr21 vary significantly, which may likely be due to large differences between personal genomes, which can result in differences in the correction of the abundance of proteins encoded on chromosome 21, similar to what happens in aneuploid yeast

Studies of the transcriptome and proteome of individual human cells at the stages of early embryogenesis (Wang S. et al., 2024) have significantly expanded the understanding of the role and mechanisms of manifestation of aneuploidy. A transcriptome analysis of about 15 thousand individual cells from 203 eu- and aneuploid human blastocysts (epiand hypoblasts, polar and mural trophectoderm) showed that changes in the copy number of chromosomes are significant for ~20 % of genes. About 90 dosage-dependent domains have been identified in aneuploid chromosomes. Especially in monosomies, common consequences like apoptosis were found, which helps to explain why autosomal monosomies occur in fewer cells. It is likely that with autosomal monosomies, critical developmental disorders occur even before implantation. Of course, the cause of such disorders may be not only or not so much a change in gene dosage but a loss of heterozygosity, leading to the absence of complete copies of some genes in the cell. In this regard, it is not surprising that the sets of dosage-dependent genes in complementary tri- and monosomies turned out to be different. The downregulation of TGF-β and FGF signaling, which led to deficient trophectoderm maturation, was another lineage-specific consequence that caused unstable epiblast formation in aneuploids (Wang S. et al., 2024).

## Aneuploidy and architecture of interphase nuclei

Previously, it was believed that changes in the copy number of chromosomes of the main set have an effect on the phenotype, mainly due to the imbalance of gene copies. However, in humans, the manifestation of a number of syndromes (at least with Tr21) is caused not only by an increased expression of genes from the aneuploid chromosome (Olson et al., 2004). Trans effects of aneuploidy have also been identified, and it has been hypothesized that the disruption of cellular homeostasis is caused by the presence of an extra chromosome (Krivega et al., 2022).

In the interphase nucleus, chromosomes and their regions are not randomly located relative to transcriptionally active and inactive compartments (Cremer T., Cremer C., 2001; Cremer T., Cremer M., 2010; Cremer M. et al., 2020). Moreover, the architectonics of the nucleus and chromosomal territories may differ both at different stages of ontogenesis and in different cell types (Croft et al., 1999; Tanabe et al., 2002). In the nuclei of cells that differ in morphology and tissue affiliation, different principles of spatial localization of chromosomes and chromosomal regions can be implemented (Cremer M. et al., 2003; Mayer et al., 2005; Solovei et al., 2013), determining its functional compartmentalization due to the specific distribution of transcriptionally active and inactive chromatin regions in the nucleus (Meaburn, Misteli, 2007).

The development of 3D genomics (3C, chromosome conformation capture, Hi-C, ChIA-PET, Micro-C, snHi-C, etc.) has significantly expanded the understanding of the levels of hierarchical and spatial organization of chromatin in the nucleus and the dynamics and plasticity of the structural and functional compartments of the nucleus (Dekker et al., 2002; Li G. et al., 2010; van Berkum et al., 2010; Nagano et al., 2013; Hsieh et al., 2020). For the human genome, topologically associated domains (TADs), A/B compartments and their subcompartments (Oji et al., 2024), chromatin loops, lamina-associated domains (LADs), nucleolus-associated domains (NADs), and their variants in different cell types and at different stages of development/differentiation are described in detail. Recent studies have investigated the influence of structural and numerical chromosomal aberrations on the spatial organization of chromatin (Shao et al., 2018; Wang Y. et al., 2023; Zhegalova et al., 2023).

The mechanisms of the influence of aneuploidy on changes in the spatial organization of chromatin in the nucleus are unknown, and in this work, we present only data from studies describing changes in nuclear architecture in aneuploid human cells. Important factors that determine the structural and functional organization of the genome are the connection of its specific sections with the nuclear lamina, the localization of chromatin relative to the nucleolus, and the formation of interchromatin compartments (nuclear bodies) (Razin, Ulianov, 2022). The spatial organization of the nucleus is determined primarily by the anchoring of chromosomal territories on the nucleolus (helped by NADs) and the nuclear lamina (helped by LADs), as well as the presence of interchromatin compartments (nuclear bodies) (Razin, Ulianov, 2022).

Although about a third of the human genome contains potential LADs (van Steensel, Belmont, 2017) in different cell types, only about 30 % of potential LADs are associated with the lamina (Zhegalova et al., 2023). Most genes located in lamina-associated regions are not expressed or expressed at low levels. Alterations in the composition of lamina-associated regions lead to changes in the transcriptome of the cell (van Steensel, Belmont, 2017; Shah et al., 2023). An important role is played by the distribution of LADs along the chromosome; chromosomes with a small proportion of LADs tend to be found medially, in the center of the nucleus; for example, human chromosome 19 is characterized by the highest gene density and has an internal position in the nucleus (Croft et al., 1999). Due to the altered chromosome copy number, the conditions of competition of potential LADs for association with the lamina may change, which can lead to changes in the structural organization of chromatin, and not only that of the aneuploid chromosome. This, in turn, can lead to changes in the transcriptional activity of genes located on different chromosomes, and such changes can be critical, leading to disorders already at early stages of development (Zhegalova et al., 2023).

As an example, we can consider the organization of chromatin in the nuclei of aneuploid human colonic epithelial cells (HCEC) with trisomy of chromosome 7. 3-D FISH, a wholechromosome probe that specifically stains the corresponding chromosomal territory did not reveal fundamental changes in the localization of the territory of the aneuploid chromosome in the interphase nucleus. However, Hi-C analysis, in addition to an increase in the frequency of interchromosomal contacts of DNA regions of chromosome 7, revealed changes in A/B compartmentalization and in the boundaries of TADs. Changes in the chromatin of chromosome 4 were detected: a reduction in the number of TADs (from 133 to 109) and movement of the chromatin of a chromosome 14 region (chr14:62.4Mb– 63.8Mb) from the active A to the inactive B compartment (Braun et al., 2019).

In human chorionic villi cells at Tr21, changes in the nuclear localization of chromosomal territories of chromosomes 1 and 3 were noted (Kemeny et al., 2018). When studying other trisomies (Tr13, Tr16 in chorion cells; Tr18 in in vitro cultured fibroblasts), changes in patterns of interchromosomal contacts were noted for all human chromosomes (Zhegalova et al., 2023). These studies revealed a correlation between the number of loci with altered compaction and the number of LADs in the aneuploid chromosome (Tr13, Tr18). It turned out that the number of LADs in chromosomes 13 and 18 is three times higher than in chromosome 16, which could potentially cause a more pronounced effect on the chromatin-lamin interactome in the nucleus, leading to changes in chromatin compaction. In addition, it turned out that the number of loci with altered compaction in small chromosomes is higher in Tr16 compared to Tr13 and Tr18. The presence of an extra chromosome 16 also significantly reduced the frequency of DNA contacts of small chromosomes (chromosomes 16–22) in chorion cells (up to 20 % for a single pair of chromosomes). The authors suggest that additional copies of small chromosomes, compe- ting with copies of similar small chromosomes, lead to changes in the distribution of their material in the nucleus, reducing the frequency of contacts (Zhegalova et al., 2023). In NPCs (neuronal progenitor cells), an extra copy of chromosome 21 increased the frequency of DNA contacts within the group of small chromosomes HSA16–22. Thus, aneuploidies of different chromosomes can lead to different changes in the spatial organization of chromatin in the interphase nucleus, and such changes can be different in different cell types (Meharena et al., 2022; Zhegalova et al., 2023).

The authors believe that in trisomies, different variants of spatial DNA contacts can be formed in different subpopulations of cells (Zhegalova et al., 2023), and the observed differences in the Hi-C data array may reflect the combined effect of several factors (the presence of an extra chromosome, the proliferative activity and age of the cell, the degree of its differentiation, etc.). Changes in the structural and functional organization of chromatin are probably of critical importance in early embryogenesis and are the cause of formation of multiple abnormalities in different tissues and organs observed in trisomy (Zhegalova et al., 2023). The authors believe that changes in the spatial organization of chromatin, systematic and stochastic, are determined by a combination of many factors, including the size of the chromosome, its LAD coverage, and the density of gene localization in it (Zhegalova et al., 2023). However, it should be recognized that most questions about the effect of aneuploidy on the architecture of the nucleus and the structural and functional organization of chromatin remain unanswered.

The small amount of research conducted, which sheds only a little light on the effect of aneuploidy on the spatial organization of the nucleus in trisomies in human cells, leaves open the question of the presence of features or general patterns in changes in the spatial organization of the genome during chromosomal aberrations

## Mosaic aneuploidy

As a result of errors that occur in mitosis during the proliferation of somatic cells, cells with an altered genome constantly appear in the body. As a result, most organisms are mosaics. In humans, aneuploid cells are present in various tissue types, including hepatocytes (2.2 %), neurons (<5 %), lymphocytes, etc. (Knouse et al., 2014). Aneuploidies of different chromosomes (HSA1, 7, 8, 9, 10, 11, 14, 15–18, 21, and X/Y) have been identified in brain cells (Graham et al., 2019). It is possible that somatic mosaicism contributes to the formation of diversity, in which neurons of the same lineage perform different functions (McConnell et al., 2017). It turned out that somatic mosaicism is more often observed for sex chromosomes than for autosomes (Machiela et al., 2016). In lymphocytes, mosaicism on the Y chromosome associated with its loss (mLOY) is the most common type of aneuploidy (1.7–20 %) (Graham et al., 2019). Several characteristics should be considered when examining mosaicism studies. Accordingly, if the percentage of cells with a different karyotype was at least 5 % when mosaicism was detected using FISH conducted on interphase nuclei, it was deemed significant (Modi et al., 2003; Yurov et al., 2007); however, 1.6 % was already deemed significant when mosaicism on the X chromosome was examined (Guttenbach et al., 1995).

The phenotype of mosaics depends on the proportion of aneuploid cells, which may vary in different tissues and at different stages of development. Analysis of individual cells of embryos at the preimplantation stage (blastocyst) showed the presence of aneuploid and mosaic embryos. According to different studies, the proportion of mosaic embryos varied from 2 to 90 % (Starostik et al., 2020; Rana et al., 2023). It is worth noting that in a number of cases, when analyzing a pool of cells, mosaicism in embryos was not detected, since aneuploidy in the cells was compensatory (trisomy and monosomy on the same chromosome in different cells). The use of methods for analyzing the genome and transcriptome of single cells (scWGS, scRNAseq) of the embryo has made it possible to describe in more detail the levels of mosaicism at different stages of embryonic development. It was found that 100 % of the analyzed embryos were mosaics at the blastocyst stage; during the development of the embryo, at later stages of its development (5–26 weeks of gestation), the proportion of aneuploid cells decreased. In addition, cases of healthy children being born with a normal karyotype, although aneuploidy was detected during retrospective analysis of their embryonic cells, have been described (Zhai et al., 2024).

Concluding a brief discussion of the problems of mosaicism associated with aneuploidy of different chromosomes, we note that it can occur in cancer cells after WGD in the early stages of tumorigenesis (Lambuta et al., 2023) and/or as a result of CIN, including both numerical and structural chromosome aberrations (Li R., Zhu, 2022). According to recent data, WGD is detected in 30 % of tumors at the early stages of tumorigenesis (Lambuta et al., 2023). Up to 90 % of solid tumors and 70 % of hematopoietic malignancies are associated with aneuploidy (Xiao et al., 2024). An increased frequency of chromosomal abnormalities, including aneuploidy, is also observed in in vitro cultured human embryonic stem cells, which may contribute to their potential tumorigenicity (Baker et al., 2007). The phenomenon of CIN, associated with WGD and/or aneuploidy, is often accompanied by genomic instability and manifests itself in the form of diversity of tumor cell karyotypes and high intra- and inter-tumor heterogeneity of the cancer cell genome (Burrell et al., 2013).

## Segmental aneuploidy and CNVs

Segmental aneuploidy and CNV might have distinct origins. In carriers of balanced translocations, unbalanced gametes arise, leading to various clinically significant forms of segmental aneuploidy. Despite the 50 % frequency of such gametes, the percentage of children with partial trisomy and partial monosomy in such parents is lower. It is unknown when selection favors carriers of a balanced genome, and it may vary depending on the type of chromosomal rearrangement. Even standard cytogenetic techniques can easily determine a balanced translocation in parents if both translocation-related chromosomal regions are relatively large. Unfortunately, detecting such a balanced translocation can be difficult if one of the chromosomal regions is small and distal. To do this, FISH using DNA probes specific to distal regions of the chromosomes or microarray-CGH is required. The goal of medical cytogenetics is to discover and characterize carriers of these combined partial trisomies and monosomies as well as carriers of balanced chromosomal translocations, whose offspring may also be carriers of combined partial trisomies and monosomies. It should be noted that these combinations have a pathogenic effect.

DNA replication and repair errors result in the loss or gain of chromosome regions, leading to CNVs and segmental aneuploidies. In studying the clinical significance of such segmental aneuploidies and CNVs, researchers have encountered unique challenges. Whole-genome sequencing of thousands of personal human genomes has revealed a huge number of bi- and multiallelic single nucleotide variants (SNVs, Single Nucleotide Variants), biallelic indels, and structural variants (SVs, Structural Variants) of the genome, including large insertions, deletions, inversions, and variations of genomic regions by copy number (The 1000 Genomes Project Consortium et al., 2015). Given this variability, assessing the potential pathogenic significance of variations in a specific genome region’s copy number frequently proves to be quite a challenging task. In this section, we will consider cases of appearance of additional copies of genome regions because, in a diploid organism, loss of a chromosomal region leads to haploidization of part of the genome and usually has a pronounced pathogenic effect or a delayed pathogenic effect. However, in the case of a tetraploid genome, the loss of one copy of a genomic fragment may be one of the first stages towards genomic rediploidization, which is a very important stage in genomic evolution, but its consideration is beyond the scope of this review.

A bioinformatics study of an aneuploid chromosome region’s composition usually involves considering a number of hypotheses. Due to the enormous genomic diversity in humans, analysis of a large number of patients is required to make a definitive conclusion about the clinical significance of specific CNVs. In addition, since the same CNVs or segmental aneuploidies can manifest themselves in fundamentally different ways in different genomes, analysis of a large number of cases of a particular CNV in relatives may not provide a definitive answer. Finding patients with identical CNVs is often a challenging task because the frequency of each specific CNV is low, and the study of patients and their relatives reduces the ability to assess its clinical significance when found in different genomes. As a result of the analysis of a large sample of patients, CNVs can be classified as either variants without pathogenic influence, or without potential pathogenic influence, or as CNVs with unknown influence on the phenotype, or as CNVs with possible potential pathogenic influence, and finally as CNVs with pathogenic influence (Zhang F. et al., 2009; Auwerx et al., 2022). It should be taken into account that the genomes of people from diverse populations have substantial differences and are well divided into clades (Mallick et al., 2016). Moreover, they may also differ in the presence of DNA that originates from other, long-extinct hominins (Neanderthals, Denisovans, etc.) (Vernot et al., 2016). Thus, it cannot be ruled out that a conclusion drawn for one group of populations will be incorrect for another.

Importance of localization of segmental duplications in the genome

The location of the chromosome’s changed copy number region is important. Duplications may occur as a single structural and functional element of the chromosome (TAD) or as a tandem cluster of duplicons, distant from the original sequence, in a human small supernumerary marker chromosome (sSMC), or in an extra chromosome (B chromosome) in other eukaryotic species. If the structural and functional organization of the duplicated region and its localization are in tandem relative to the original region and are preserved, one can expect the presence of transcriptional activity of the genes included in this region.

It is more difficult to assess the impact of additional material in human sSMC due to their variable content. The majority of them are composed of the original chromosome’s pericentromeric region, which includes nearby heterochromatin and perhaps euchromatin with a variable number of genes. It has been observed that if the size of the euchromatic region of human sSMC does not exceed 3–5 Mb, it usually does not have a pathogenic effect. It can be assumed that the absence of negative phenotypic traits in the carrier of such sSMC is associated with inactivation of the sSMC material due to the localization of its domains in the transcriptionally inactive compartment of the interphase nucleus in comparison with the homologous region of the original chromosome. Therefore, conducting a number of studies on the spatial organization of the genome with sSMCs of varying sizes and DNA content is an urgent and highly intriguing task, the resolution of which would enable us to assess the potential pathogenic effect of different sSMCs.

## Conclusion

Considering the data on the manifestation of various variants of aneuploidy, it should be noted that there are a huge number of factors that can play a very significant role and influence their manifestation. A significant factor is probably the complexity of the tissue and organ organization of the organism of a particular species. Thus, yeast, like cell cultures, is quite tolerant of chromosomal aneuploidy. Polyploid organisms and species that have relatively recently undergone whole-genome duplication are usually much more resistant to aneuploidy. A special position is occupied by aneuploidy of sex chromosomes, which may be due to the peculiarities of their gene composition formed during the process of evolution.

A special variant of aneuploidy is represented by segmental aneuploidies and CNVs. In these cases, the composition of the additional material, its localization in the chromosome, and its localization in different compartments of the interphase nucleus may be of particular importance. Of particular interest are the mechanisms of dosage compensation for changes in the level of gene product during aneuploidy at the posttranscriptional and post-translational levels.

The study of aneuploidies and their clinical significance is of great interest in light of data on the huge diversity of personal human genomes, including SNVs, SVs, and CNVs. It can make a great contribution to improving cytogenomic diagnostics by creating the necessary database for the correct interpretation of identified cases of CNVs and segmental aneuploidy.

## Conflict of interest

The authors declare no conflict of interest.
